# 5-Fluorouracil targets thymidylate synthase in the selective suppression of T_H_17 cell differentiation

**DOI:** 10.18632/oncotarget.8344

**Published:** 2016-03-24

**Authors:** Juan Wang, Liang Peng, Ruihua Zhang, Zihan Zheng, Chun Chen, Ka Lung Cheung, Miao Cui, Guanglin Bian, Feihong Xu, David Chiang, Yuan Hu, Ye Chen, Geming Lu, Jianjun Yang, Hui Zhang, Jianfei Yang, Hongfa Zhu, Shu-hsia Chen, Kebin Liu, Ming-Ming Zhou, Andrew G. Sikora, Liwu Li, Bo Jiang, Huabao Xiong

**Affiliations:** ^1^ Guangdong Provincial Key Laboratory of Gastroenterology, Department of Gastroenterology, Nanfang Hospital, Southern Medical University, Guangzhou, China; ^2^ Department of Medicine, Immunology Institute, Icahn School of Medicine at Mount Sinai, New York, NY, USA; ^3^ Department of Biology, College of Arts and Sciences, University of North Carolina at Chapel Hill, NC, USA; ^4^ Amgen Inc., Thousand Oaks, CA, USA; ^5^ Department of Biological Sciences, Virginia Tech, Blacksburg, VA, USA; ^6^ Department of Structural and Chemical Biology, Icahn School of Medicine at Mount Sinai, New York, NY, USA; ^7^ Department of Pathology, Icahn School of Medicine at Mount Sinai, New York, NY, USA; ^8^ Tempero Pharmaceuticals, GlasxoSmithKline, Cambridge, MA, USA; ^9^ Department of Biochemistry and Molecular Biology, Medical College of Georgia, Georgia Regents University, Augusta, GA, USA

**Keywords:** 5-FU, TH17, TS, Immunology and Microbiology Section, Immune response, Immunity

## Abstract

While it is well established that treatment of cancer patients with 5-Fluorouracil (5-FU) can result in immune suppression, the exact function of 5-FU in the modulation of immune cells has not been fully established. We found that low dose 5-FU selectively suppresses T_H_17 and T_H_1 cell differentiation without apparent effect on Treg, T_H_2, and significantly suppresses thymidylate synthase (TS) expression in T_H_17 and T_H_1 cells but has a lesser effect in tumor cells and macrophages. Interestingly, the basal expression of TS varies significantly between T helper phenotypes and knockdown of TS significantly impairs T_H_17 and T_H_1 cell differentiation without affecting the differentiation of either Treg or T_H_2 cells. Finally, low dose 5-FU is effective in ameliorating colitis development by suppressing T_H_17 and T_H_1 cell development in a T cell transfer colitis model. Taken together, the results highlight the importance of the anti-inflammatory functions of low dose 5-FU by selectively suppressing T_H_17 and T_H_1 immune responses.

## INTRODUCTION

Interleukin (IL)-17 producing T helper cells (T_H_17), are a T helper subtype clearly distinct from T-helper type 1 (T_H_1), T-helper type 2 (T_H_2), and T regulatory (Treg) cells, which play an important role in the pathogenesis of various inflammatory and autoimmune diseases [1, 2]. T_H_17 cell differentiation, survival, and expansion depend on many cytokines and transcription factors that work in concert to drive the induction of unique program. Transforming growth factor β (TGF-β) and Interleukin-6 (IL-6) have been described as key factors contributing to generating de novo T_H_17 cells [3-5], but other cytokines have been shown to have significant auxillary functions as well: IL-23 is necessary for T_H_17 lineage expansion [2, 6], and IL-21 is also involved the differentiation of T_H_17 cells [7-9]. T_H_17 cells secrete several recognized signature cytokines including IL-17A, IL-17F and IL-22 [2, 10-13]. The retinoic acid receptor-related orphan nuclear receptor (RORγt) has been identified as a critical transcription factor for T_H_17 cell differentiation [14] while several other transcription factors including RORα, STAT3, and IRF4 have been reported to be involved [1, 14-16]. Other metabolism-related factors have also been implicated in regulating T_H_17 differentiation, and have been the focus of significant recent studies [17, 18]. For instance, the NFAT5 pathway has been shown to upregulate T_H_17cells in response to high salt [19], and the deprivation of free amino acids has been reported to bias against T_H_17 differentiation [20].

Accumulating experimental evidence indicates that T_H_17 cells are indeed involved in the pathogenesis of various autoimmune/inflammatory diseases [1]. Yen at al demonstrated that T_H_17 cells are responsible for the development of colitis in a murine colitis model [21]. Leppkes et al indicated that RORγt-expressing T_H_17 cells play a critical role in intestinal inflammation [22]. Fuss et al. demonstrated that T_H_17 cell-inducing IL-23 was highly expressed in active Crohn's patients [23]. However, recent reports have indicated that IL-17A plays a protective role in T cell-mediated intestinal inflammation [24] and a clinical trial showed that treatment with anti-IL-17A antibodies was ineffective for IBD patients [25]. As such, the exact mechanisms for the T_H_17 cells contributing to the development of inflammatory diseases still need to be further explored.

5-FU, an analogue of uracil, is commonly used as a chemotherapeutic drug for treating various kinds of tumors, primarily through inhibition of TS [26-29]. Due to its structural similarity to uracil, 5-FU and its metabolites are readily taken up by many cells. Once inside the cell, 5-FU can either bind to TS or otherwise be incorporated into the dNTP pool [27]. From the nuclear dNTP pool, 5-FU may induce DNA damage during replication, and hinder DNA repair [27]. 5-FU can induce apoptosis in cells over time as a result [28, 30]. In colorectal cancer (CRC), 5-FU's efficiency has been linked to cancer cells being strongly affected by thymidine starvation and DNA damage due to higher rates of replication [17, 31]. As part of the FOLFOXIRI treatment regimen, patients may receive 1000mg/m^2^ of 5-FU over 24 hrs to 3200 mg/m^2^ over 48 hours, and similar amount in the older FOLFIRI regimen [32, 33]. 5-FU may also be provided at 425mg/m^2^ as part of adjuvant therapy [34]. Clinically, it is apparent that cancer treatment with 5-FU results in the unfortunate side effect of broad immune suppression [23, 27]. However, the mechanism(s) by which 5-FU modulates immune cells have not been fully understood.

In our present study, we demonstrate that low dose 5-FU selectively suppresses T_H_17 and T_H_1 cell differentiation without major effects on Treg, T_H_2 cells. This effect is not seen with high dose 5-FU, suggesting clinical usage and low doses of 5-FU may have different immunomodulatory effects. These results highlight the importance of anti-inflammatory functions of low dose 5-FU by selectively suppressing T_H_17 and T_H_1 immune responses both *in vitro* and *in vivo*.

## RESULTS

### Low dose 5-FU selectively suppresses T_H_17 and T_H_1 cell differentiation

**Figure 1:**
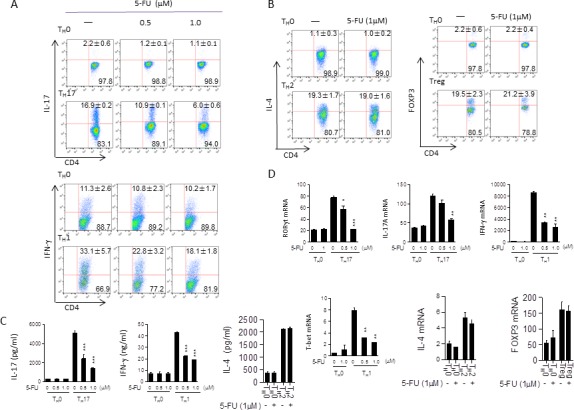
Low dose 5-FU selectively suppresses T_H_17 and T_H_1 cell differentiation while has no major effects on T_H_2 and Treg cell differentiation **A.** Naïve CD4^+^ T cells from C57BL/6 mice were differentiated under T_H_17 and T_H_1 polarizing conditions respectively in the presence of 5-FU (0.5, 1.0 μM) for 3 days and analyzed through flow cytometry. **B.** Naïve CD4^+^ T cells from C57BL/6 mice were differentiated under T_H_2 and Treg polarizing conditions respectively in the presence of 5-FU (1.0 μM) for 3 days and analyzed through flow cytometry. **C.** Supernatants from cells cultured in (A) and (B) analyzed *via* ELISA. **D.** Cells cultured as in (A) and (B) for 48 hours; mRNA expression of the indicated genes was determined by qPCR. *p < 0.05, **p < 0.01, ***p < 0.001 *versus* cells cultured without 5-FU.

5-FU is a first-line chemotherapeutic drug for treating CRC, which can result in the side effect of immunosuppression. However, the exact mechanism by which 5-FU suppresses immune responses remains unclear. To investigate the effects of 5-FU on immune cell function, we first focused on T helper cells. Naïve CD4^+^ T cells from C57BL/6 mice were primed *in vitro* for 3 days under T_H_0, T_H_17, T_H_1, T_H_2, or Treg polarizing conditions in the presence of 5-FU at different concentrations. Interestingly, the frequency of IL-17- and IFN-γ-producing cells (IL-17^+^ cells from 16.9% to 6.0%; IFN-γ^+^ cells from 33.1% to 18.1%) decreased following 5-FU treatment in a dose-dependent manner, suggesting that 5-FU may have a selective effect (Figure 1A). These observations correlated with reduced IL-17 and IFN-γ production by T_H_17 or T_H_1 cells treated with 5-FU as determined by ELISA (Figure 1C). Interestingly, T_H_2, Treg, T_H_9, and T_H_22 differentiation were not noticeably affected in T cell cultures treated with 5-FU at that lower dosage (Figure 1B, 1C, 1D, Supplementary Figure 1A, 1B, 1C, 1D). Furthermore, qPCR experiments showed low dose 5-FU significantly suppressed mRNA expression of T_H_17 or T_H_1-associated genes including IL-17, RORγt, IFN-γ, and T-bet (Figure 1D).

To rule out the possibility that the reduced T_H_17 and T_H_1 cell differentiation was due to abnormal cell death caused by 5-FU, we analyzed CD4^+^ T cells from spleens as well as lymph nodes of C57BL/6 mice and tumor cell lines. Using Annexin V and PI staining for cell death, we tested a range of concentrations of 5-FU on naïve T cells and tumor cells. T cells were sensitive to 5-FU and as the concentration causing clear T cell death is 2.5 μM, while the concentration of 5-FU inducing tumor cell death is 20 μM (Supplementary Figure 2A, 2B). Since 5-FU just caused minimal cell death in naïve T cells up to a concentration of 1 μM, we set that as our working dose in our subsequent investigations (Supplementary Figure 2A). Notably, this dose is much lower than that used clinically, and did not lead to tumor cell death (Supplementary Figure 2B). Furthermore, 5-FU had no significant effect on the expression of IL-10 (Supplementary Figure 3). Thus, the decreased T_H_17 and T_H_1 cell differentiation induced by 5-FU was not due to the alterations on IL-10 levels.

### 5-FU alters DNA binding activity in T_H_17 and T_H_1 cells

**Figure 2:**
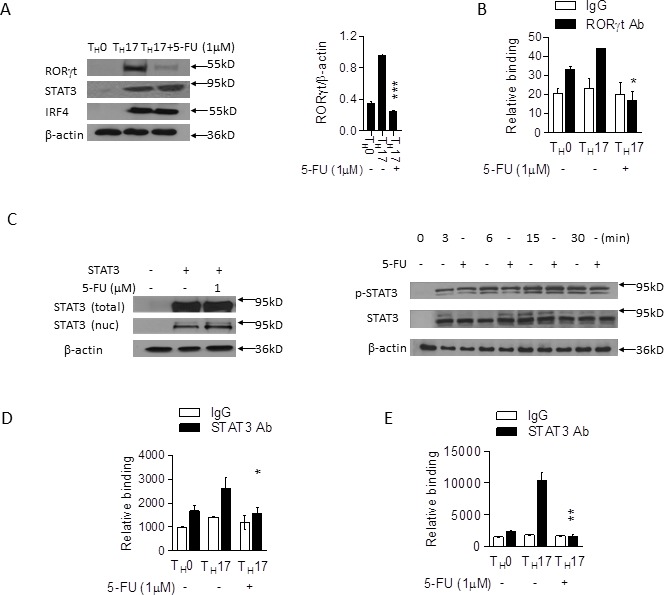
5-FU alters STAT3 DNA binding activity in T_H_17 cells **A.** Naïve CD4^+^ T cells from C57BL/B6 mice cultured in T_H_17 polarizing conditions in the presence of 5-FU (1.0 μM) for 3 days prior to western blotting for RORγt and other indicated proteins. **B.** The same cells were cultured for 60 hours, followed by ChIP assay. 3 μg of anti- RORγt antibody or isotype-matched IgG control antibody were used in the immunoprecipitation step. qPCR was used to quantify the amount of precipitated DNA with primers flanking the RORγt binding the CNS2 of the IL-17 promoter region. **C.** 293T cells transfected with STAT3 plasmid for 40 hours in the presence of 5-FU (1 μM). The cytosolic fraction and nuclear fraction proteins were analyzed *via* western blotting. Cells were cultured as in (A), with western blotting for STAT3 protein phosphorylation and STAT3 protein expression at the times indicated. Cells treated as in (A) analyzed *via* ChIP assay. 3 μg of anti-STAT3 antibody or isotype-matched IgG as control antibody were used in the immunoprecipitation step. qPCR was used to quantify the amount of precipitated DNA with primers flanking the STAT3 binding site of the RORγt **D.** and the CNS2 of the IL-17 (E) promoter region. *p < 0.05, **p < 0.01, ***p < 0.001 *versus* cells cultured without 5-FU.

The data above prompted us to probe for the molecular basis for which 5-FU modulates T_H_17 cell differentiation. Since many studies have shown that several transcription factors including RORγt, STAT3, and IRF4 are important for T_H_17 cell differentiation [23], we hypothesized that low dose 5-FU might affect the expression of these transcription factors. To address this, naïve CD4^+^ T cells from C57BL/6 mice were primed *in vitro* for 3 days under T_H_0 or T_H_17 polarizing conditions. Western blotting experiments showed that the protein expression of RORγt was significantly reduced in the cells treated with low dose 5-FU (Figure 2A). However, the levels of STAT3 and IRF4 protein were comparable in the presence or absence of low dose 5-FU (Figure 2A). In addition, ChIP analysis demonstrated that the binding of RORγt to the promoter region of IL-17 gene was significantly reduced (Figure 2B). Since STAT3 is important for RORγt expression, we next analyzed the effects of 5-FU on STAT3 activation. Western blotting showed that 5-FU did not affect the levels of STAT3 expression (Figure 2A, 2C) or nuclear translocation (Figure 2C), or STAT3 phosphorylation (Figure 2C). However, ChIP experiments showed that the binding of STAT3 to the promoter region of RORγt gene was significantly reduced (Figure 2D, 2E), suggesting that 5-FU inhibits STAT3-mediated activation, leading to the suppression of T_H_17 cell differentiation.

**Figure 3:**
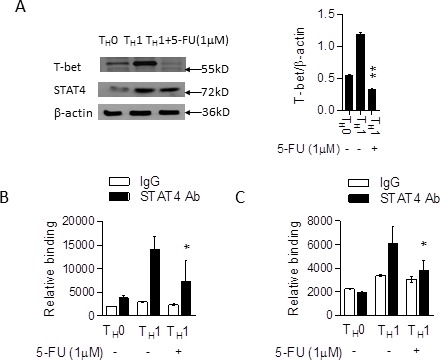
5-FU alters STAT4 DNA binding activity in T_H_1 cells **A.** Naïve CD4^+^ T cells from C57BL/6 mice were differentiated under T_H_1 polarizing conditions in the presence of 5-FU (1.0 μM) for 3 days and were analyzed by western blotting. **p < 0.01 *versus* cells not added with 5-FU. Naïve CD4^+^ T cells from C57BL/6 mice were cultured under T_H_1-polarizing conditions in the presence of 5-FU (1.0 μM) for 60 hours, followed by ChIP assay. 3 μg of anti-STAT4 antibody or isotype-matched IgG as control antibody were used in the immunoprecipitation step. qPCR was used to quantify the amount of precipitated DNA with primers flanking the STAT4 binding site of the T-bet **B.** and the IFN-γ **C.** promoter region. *p < 0.05 *versus* cells cultured without 5-FU.

To investigate the molecular mechanism by which 5-FU affects T_H_1 cell differentiation, we first examined the effects of 5-FU on the expression of STAT4 and T-bet, key transcription factors for T_H_1 cells. Western blotting showed that 5-FU had no clear effect on STAT4 expression (Figure 3A). However, T-bet protein expression was significantly reduced in T_H_1 cells in the presence of 5-FU (Figure 3A), suggesting that 5-FU inhibits T-bet expression leading to the suppression of T_H_1 cell development. Since STAT4 is important for T-bet expression, we next questioned if 5-FU influences STAT4 activation. ChIP experiments showed that the binding of STAT4 to the promoter region of T-bet gene was significantly reduced with 5-FU treatment (Figure 3B, 3C), suggesting that 5-FU inhibits STAT4 DNA binding activity resulting in the suppression of T-bet expression and T_H_1 cell development.

**Figure 4:**
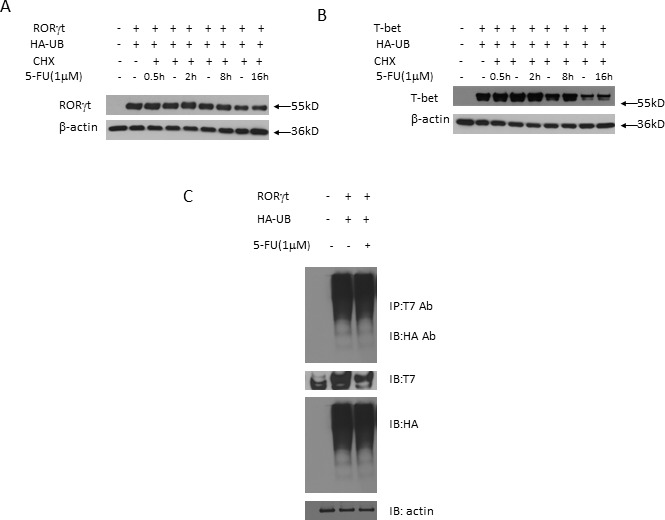
The suppression of 5-FU on T_H_17 and T_H_1 has no relation to proteasome proteolytic pathway **A.** 293T cells were co-transfected with HA-tagged ubiquitin (HA-Ub) and T7-tagged RORγt plasmids in the presence of cycloheximide (CHX) and 5-FU (1 μM) for various time intervals and the cell lysates were collected and RORγt protein expression was analyzed by western blotting. **B.** 293T cells were co-transfected with HA-UB and T-bet plasmids in the presence of CHX and 5-FU (1 μM) for various time intervals and the cell lysates were collected and T-bet protein expression was analyzed by western blotting. **C.** 293T cells were transfected with T7-tagged RORγt and HA-Ub overexpression plasmids for 36 hrs. The cell lysates were immunoprecipitated with anti-T7 antibody and immunoblotted with anti-HA antibody. The results are representative of three independent experiments.

We then proceeded to analyze whether 5-FU modulates posttranslational modification of RORγt or T-bet protein resulting in proteasome-mediated protein degradation. To test this, we co-transfected T7-RORγt, T-bet and HA-tagged ubiquitin overexpression plasmids into 293T cells added with 5-FU. 5-FU treatment had no significant effect on RORγt or T-bet protein degradation or ubiquitination (Figure 4A, 4B, 4C), suggesting that 5-FU is not involved in the control of RORγt and T-bet protein stability and ubiquitination.

### 5-FU inhibits TS expression in T_H_17 and T_H_1 cells

**Figure 5:**
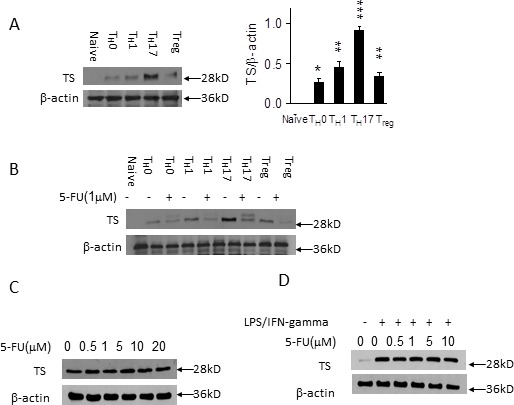
5-FU inhibits TS expression in T_H_17 and T_H_1 cells **A.** Naïve CD4^+^ T cells from C57BL/B6 mice were differentiated under T_H_0, T_H_1, T_H_17, and Treg polarizing conditions respectively for 3 days. The expression of TS protein was analyzed by western blotting. *p < 0.05, **p < 0.01, ***p < 0.001 *versus* control cells. **B.** Cells cultured as in (A) in the presence of 5-FU (1 μM) for 3 days. **C.** SW620 were cultured in the presence of 5-FU (0.5, 1.5, 10, 20 μM) for 72 hours for western blotting, **D.** BMDMs were stimulated with IFN-gamma (10 ng/ml) and LPS (200 ng/ml) in the presence of 5-FU (0.5, 1.5, 10 μM) for 24 hours for western blotting.

**Figure 6:**
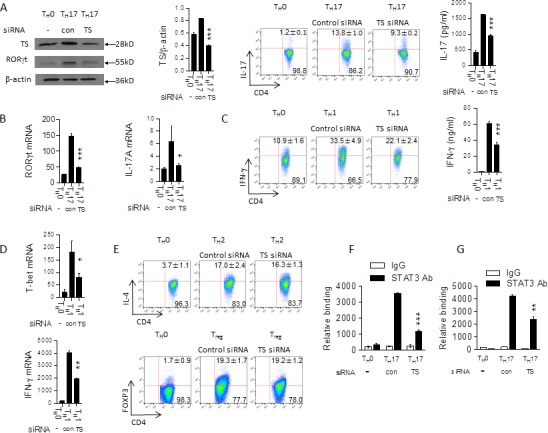
Knockdown of the TS suppresses T_H_17 and T_H_1 cell differentiation **A.** Naïve CD4^+^ T cells from C57BL/6 mice were transfected with TS siRNA or control siRNA and differentiated under T_H_17 polarizing conditions for 72 hours. Cell lysates were analyzed by western blotting. The cells were cultured for 72 hours and then were re-stimulated with PMA/ionomycin for 5 hours, stained for intracellular IL-17, and analyzed by flow cytometry, and the supernatants were analyzed for IL-17 by ELISA. ***p < 0.001 *versus* cells transfected with control siRNA. **B.** Cells treated as in (A) for 48 instead of 72 hours; mRNA expression of indicated genes was determined by qPCR. *p < 0.05,***p < 0.001 *versus* cells transfected with control siRNA. **C.** Naïve CD4^+^ T cells from C57BL/6 mice were transfected with TS siRNA or control siRNA and differentiated under T_H_1 polarizing conditions for 72 hours and analyzed by flow cytometry and the supernatants were analyzed for IFN-γ by ELISA. ***p < 0.001 *versus* cells transfected with control siRNA **D.** Cells as in (C) cultured for 48 hours; mRNA expression of indicated genes was analyzed by qPCR. *p < 0.05, **p < 0.01 *versus* cells transfected with control siRNA. **E.** Naïve CD4^+^ T cells from C57BL/6 mice were transfected with TS siRNA or control siRNA and differentiated under T_H_2 and Treg polarizing conditions for 72 hours and analyzed by flow cytometry. Cells treated as in (A) analyzed *via* ChIP assay. 3 μg of anti-STAT3 antibody or isotype-matched IgG as control antibody were used in the immunoprecipitation step. qPCR was used to quantify the amount of precipitated DNA with primers flanking the STAT3 binding site of the RORγt **F.** and the CNS2 of the IL-17 **G.** gene promoter region. **p < 0.01, ***p < 0.001 *versus* cells transfected with control siRNA.

Since it is well-known that 5-FU can bind directly to TS, we next investigated if that binding played a role in 5-FU's selection against T_H_1 and T_H_17 cells. Western blotting showed that protein expression of TS varies significantly between T helper phenotypes, with T_H_17 cells having the highest expression (Figure 5A). TS expression increased in all subtypes over the course of polarization, and decreased in all phenotypes in the presence of 5-FU (Figure 5B). Interestingly, 5-FU presence had no significant effect on TS expression in tumor cells (Figure 5C) and macrophages (Figure 5D). To understand the function of TS on T helper cell differentiation, we knocked down TS by transfecting TS siRNA in naïve T cells and then stimulating them under T_H_17, T_H_1, T_H_2, or Treg polarizing conditions. The results showed that knockdown of TS significantly suppressed T_H_17 (IL-17^+^ cells from 13.8% to 9.3%) and T_H_1 (IFN-γ^+^ cells from 33.5% to 22.1%) cell polarization without apparent effects on T_H_2 or Treg cell development (Figure 6A, 6B, 6C, 6D, 6E). ChIP experiments suggest TS may affect STAT3 DNA binding activity (Figure 6F, 6G). This result further demonstrates that TS itself has a role in controlling T_H_17 differentiation, ruling out the possibility that 5-FU was merely incorporating itself into DNA to induce damage. Taken together, the results suggest that low dose 5-FU selectively suppresses T_H_17 and T_H_1 cell differentiation through inhibition of TS expression.

**Figure 7:**
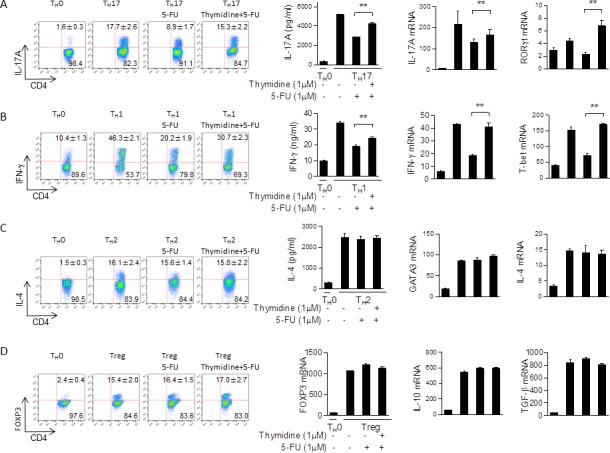
5-FU inhibition is rescued by thymidine **A.** Naïve CD4^+^ T cells from C57BL/6 mice were differentiated under T_H_17 polarizing conditions in the presence of 5-FU (1.0 μM) and thymidine (1.0 μM) for 3 days and then were re-stimulated with PMA/ionomycin for 5 hours, stained for intracellular IL-17, and analyzed by flow cytometry as shown. Supernatants from the cells were saved and tested *via* ELISA. The cells cultured for 48 hours and mRNA expression of the indicated genes was determined by qPCR. **B.** Repetition of (A) using T_H_1 polarizing conditions. **C.** Repetition of (A) and (B), except using T_H_2 polarizing conditions. **D.** Repetition of (A), (B), and (C) with Treg polarizing conditions instead. **p < 0.01, ***p < 0.001 *versus* cells without thymidine.

Given our previous results, we then performed further experiments to clarify the role of TS in T_H_17 and T_H_1 cells. In particular, we sought to understand if the suppression of T_H_17 and T_H_1 cells that correlated with decreased expression of TS was due to the loss of TS enzymatic activity. We isolated CD4^+^ T cells and polarized them with or without 5-FU in the presence or absence of thymidylate. Since thymidylate is a product of TS activity, we reasoned that it may compensate for loss of TS enzymatic activity. Our results indicate that thymidylate is indeed capable of rescuing T_H_17 (IL-17^+^ cells from 8.9% to 15.3%) and T_H_1 (IFN-γ^+^ cells from 20.2% to 30.7%) polarization suppressed by 5-FU (Figure 7A, 7B). These results were further confirmed *via* qPCR and ELISA (Figure 7A, 7B). Interestingly, T_H_2 and Treg populations were not significantly affected by thymidylate (Figure 7C, 7D). As such, it is clear that the enzymatic role played by TS is a vital component of T_H_1 and T_H_17 polarization.

### 5-FU modulates T_H_17 cell differentiation *in vivo*

**Figure 8:**
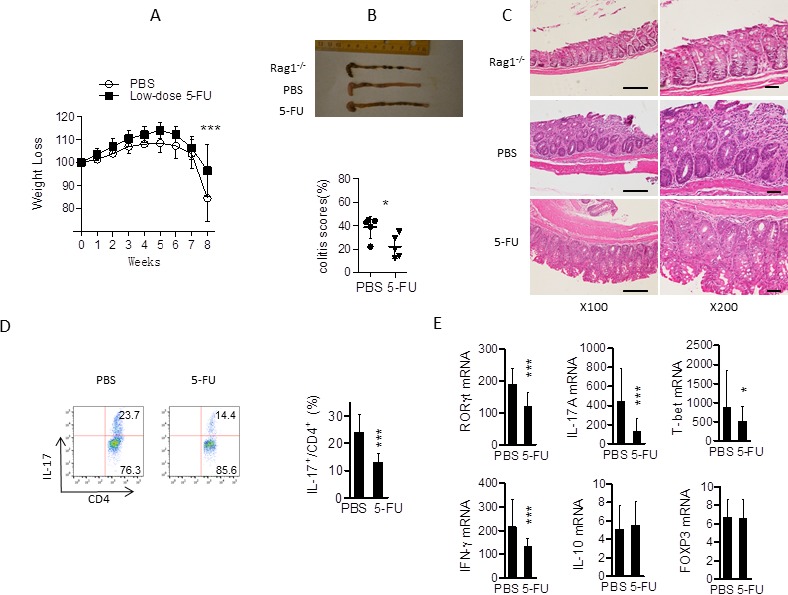
Low dose 5-FU modulates T_H_17 cell differentiation *in vivo* CD4^+^CD45Rb^hi^ T cells were purified from C57BL/6mice and 5 × 10^5^ cells were injected (i.p.) into recipient *Rag1*^−/−^ mice. Mice were treated with PBS or 5-FU (at 10 mg/kg body weight) every three days. Body weight change was monitored every week for 8 weeks. **A.** Changes in body weight of *Rag1*^−/−^ mice (*n* = 5-6 mice per group) after transfer were recorded. ***p < 0.001 *versus* recipients of PBS treated group. **B.** Morphology of intestines and disease scores, *p < 0.05 *versus* recipients of PBS group; **C.** sections of colons with colitis from *Rag1*^−/−^ mice (*n* = 5-6 mice in each group) 8 weeks after naïve T cell transfer as described above. Scale bar, 100 μM. **D.** The percentage of IL-17 -producing cells from mesenteric lymph nodes of *Rag1*^−/−^ mice in PBS and 5-FU treated group. **p < 0.01 *versus* recipients of PBS treated group. **E.** Related mRNA expression of colon was determined by qPCR. *p < 0.05, *versus* recipients of PBS treated group.

T_H_17 cells are believed to play critical roles in the pathogenesis of various autoimmune/inflammatory diseases, including multiple sclerosis (MS), inflammatory bowel disease (IBD), and rheumatoid arthritis (RA). Previous reports have indicated a correlation between T_H_17 activity and IBD pathogenesis. To further assess the effects of 5-FU on T_H_17 cell development *in vivo*, we performed adoptive T cell transfer colitis experiments using CD4^+^CD45Rb^hi^ cells from C57BL/6 mice to induce colitis in *Rag1*^−/−^ mice. Mice in the treatment group received low dose 5-FU twice a week for 8 weeks while the control group was treated with PBS. While the *Rag1*^−/−^ mice reconstituted with naïve CD4^+^ T cells lost weight continuously, treatment with 5-FU significantly improved their condition (Figure 8A). Parallel histologic studies of colonic sections from *Rag1*^−/−^ mice treated with 5-FU revealed fewer inflammatory cell infiltration and significantly lower pathological scores [35] compared to mice treated with PBS (Figure 8B, 8C). In addition, mice treated with 5-FU had significantly lower percentages of IL-17 cells and lower expression of T_H_17 and T_H_1 signature gene expression than PBS treated mice (Figure 8D, 8E).

To investigate the toxicity of high and low doses of 5-FU on intestinal mucosa, C57BL/6 mice were treated with either high dose 5-FU (50 mg/kg), low-dose 5-FU (10 mg/kg), or PBS as described above. As reported previously, high dose 5-FU significantly induced damage to intestinal mucosa; however, low dose 5-FU had no significant toxicity on intestinal mucosa (Supplementary Figure 4A, 4B), further confirming the different effect of high and low dose 5-FU on intestinal mucosa.

## DISCUSSION

Chemotherapeutic drugs that are commonly used to treat cancer affect not only tumor cells but also immune cells, having a crucial impact on antitumor responses and disease outcome [27]. Although chemotherapeutics combat tumors and lead to their regression, the effects of chemotherapeutic drugs on the tumor microenvironment and the immune cells are not fully understood. In the present study, we found that low dose 5-FU selectively suppresses T_H_17 and T_H_1 cell differentiation without major effect on Treg, T_H_2, T_H_9, T_H_22 cells, suggesting high and low dose 5-FU may have different effects on immune responses. Furthermore, we found that 5-FU inhibits STAT3 binding to the promoter regions of RORγt leading to the suppression of RORγt and IL-17 expression. In addition, 5-FU also suppresses the binding of STAT4 to the promoter region of T-bet, resulting in the modulation of T_H_1 cells. Low dose 5-FU significantly suppresses TS expression in T_H_17 and T_H_1 cells but it has no effect on TS expression in tumor cells and macrophages. Knockdown of TS in T cells resulted in the suppression of T_H_17 and T_H_1 cell differentiation and thymidylate can rescue T_H_17 and T_H_1 polarization suppressed by 5-FU. Finally, low dose 5-FU targeting TS is effective in ameliorating colitis development by suppressing T_H_17 and T_H_1 cell development in a T cell transfer colitis model. These results suggest that low dose 5-FU selectively suppressing T_H_17 and T_H_1 immune responses and highlight the potential of the anti-inflammatory activity of low dose 5-FU.

5-FU is an antimetabolite that kills rapidly proliferating cells at clinically used doses by inhibiting TS, which limits thymidine availability for DNA synthesis [27, 29, 30]. Since 5-FU can induce cell death of many tumor cell lines, 5-FU alone or in combination with other drugs is used to treat cancer in a variety of tissues including breast, cervix, colon, stomach, head and neck [36], etc. However, in addition to killing tumor cells, clinic dose of 5-FU can also eliminate other proliferating cells in the bone marrow rapidly resulting in immune suppression. Various reports have shown that 5-FU can induce cell death of immune cells including T cells, B cells and myeloid-derived suppressor cells. These results indicate that 5-FU is toxic to immune cells at certain concentrations. However, it is still not clear what kinds of effects 5-FU will induce at lower concentrations. In the present study, we demonstrate that 5-FU at low doses not induce T cell death, yet selectively suppresses T_H_17 and T_H_1 cell differentiation. Taken together, the results suggest that low dose 5-FU may play a useful anti-inflammatory role by selectively suppressing T_H_17 and T_H_1 cells.

As mentioned previously, 5-FU is a small molecule with two well-defined mechanistic pathways: being able to irreversibly inhibit TS as well as substituting as uracil to prevent effective replication, transcription and translation [27]. Our results strongly suggest that 5-FU's ability to inhibit TS is at least responsible for its selective suppression of T_H_17 and T_H_1 cell differentiation at low doses. After all, we demonstrate that TS is highly expressed by those two T effector cell types, and that such expression is conducive to their development. These results, however, do not preclude the uracil-mimic function of 5-FU from also being significant. Previous reports have demonstrated that different T helper subtypes may have different rates of metabolism, transcription, and translation [17, 18, 31, 37-40]. In particular, T effectors such as T_H_17 and T_H_1 were shown to have higher metabolism and transcription than Tregs. Given that more active cell types would also be more susceptible to mimic-induced instability, it is also possible that 5-FU may be selecting against T_H_17 and T_H_1 cells through that manner to some extent. While it is theoretically possible that T_H_17 and T_H_1 cells have higher rates of 5-FU incorporation into their DNA, such an explanation seems unlikely given the unremarkable nature of the promoters of key T_H_17 and T_H_1 proteins as compared to T_H_2 and Treg gene promoters. It may also be possible that high TS expression is indicative of the higher overall metabolism within T_H_17 and T_H_1 cells, since TS is in the salvage pathway, and not the classical purine catabolism pathway.

However, given the significant shift in TS expression that followed from the addition of 5-FU to T_H_17 and T_H_1 cells, TS is the more likely responsible for changes within those cells. If the selective effect of 5-FU on T_H_1 and T_H_17 cells was primarily a result of induced RNA and DNA damage, the rate of apoptosis would be much higher than observed. After all, other studies have demonstrated that 5-FU's tumorcidal and general immunosuppressive abilities are a result of induced cell death [41-43]. The clinical dosage used in such studies, however, is over 10 times greater than that of our estimated threshold. At such high levels, large amount of 5-FU will enter the nuclear dNTP pool, and directly interfere with replication/transcription/translation. The inhibiting effect of 5-FU on TS, while also present, would be completely overshadowed. It may well be that at low levels of 5-FU, the molecule preferentially binds to TS over joining the dNTP pool. Our future experiments will further confirm this hypothesis.

It is well-known that STAT3 is critical for RORγt and IL-17 gene expression. In the present study, we demonstrated that 5-FU significantly suppressed inhibited STAT3 DNA binding activities in T_H_17 cells. In addition, knocking-down TS in T cells significantly reduced STAT3 DNA binding activity. The results suggest that 5-FU-TS axis is clearly involved in the development of T_H_17 cells. However, it is still not clear how 5-FU-TS axis regulate STAT3 activation. Our future study will focus on how the 5-FU-TS axis regulates STAT3 protein expression, phosphorylation and translocation.

Recently, several reports demonstrated that 5-FU selectively depletes myeloid derived suppressor cells (MDSC), a population of immature myeloid cells that can suppress T cells function [44-47]. Bruchard et al has shown that high dose 5-FU activates NLRP3-dependent inflammasome activity in MDSCs resulting in the production of IL-1β which can induce secretion of IL-17 by CD4^+^ cells [47]. Interestingly, we found that low dose 5-FU (10 mg/kg) did not induce inflammasome activation in MDSCs leading to induction of IL-1β compared with high dose (50 mg/kg) in the same tumor-bearing mouse model (Supplementary Figure 5A, 5B, 5C, 5D). In addition, low dose 5-FU was not able to induce inflammasome activation in macrophages and dendritic cells (Supplementary Figure 5E). Our future studies will address the difference at the molecular levels between clinical usage and low dose of 5-FU in the modulation of immune cells.

As our results demonstrate, 5-FU is effective at selecting against T_H_17 and T_H_1 cell development *in vivo* as well, leading to significant improvement in the condition of mice in colitis models. Given the increasing prevalence and paucity of treatment options for IBD, it may serve as a useful option to consider. 5-FU has the advantage of already being fairly well characterized in terms of potential toxicological effects and of being abundantly available. As such, one might also consider additional possibilities for the use of 5-FU in treating other immune disorders as well. For instance, dysregulated T_H_17 and T_H_1 immune responses have also been implicated to be of importance in MS and RA, and it may be that 5-FU could similarly ameliorate these conditions through T_H_17 and T_H_1 suppression. Other cell types that are mainly dependent on TS expression may also be suppressed with 5-FU treatment. Overall, our results demonstrate the potential therapeutic value of using a lower dosage of a broad immunosuppressant to uncover its selective ability and report a difference in the importance of the TS pathway among T helper cells that may also be exploitable through other strategies.

## MATERIALS AND METHODS

### Mice

C57BL/6J and *Rag1*^−/−^ mice were obtained from Jackson laboratory and maintained in the barrier facility at the Icahn School of Medicine at Mount Sinai. To induce LLC tumor-bearing mice model, 5 × 10^5^ cells LLC cells were injected subcutaneously into mice. The animal study protocols were approved by the Institutional Animal Care and Use Committees of Icahn School of Medicine at Mount Sinai and Medical College of Georgia.

### Reagents and antibodies

5-FU (F6627) and Thymidine (T1895) were purchased from Sigma-Aldrich. The following Flow cytometry antibodies were purchased from BD-Biosciences (USA), and conjugated to FITC, PE, PE-Cy5, PE-Cy7, PerCP-Cy5.5, PerCP-eFluor 710, eFluor 450 or APC: CD45RB (C363.16A), CD4 (L3T4), CD25 (PC61.5), CD44(1M7), CD62L(MEL-14), CD11b (M1/70), IL-17 (TC11-18H10), IFN-γ (XMG1.2), FOXP3 (FJK-16S) and isotype controls. Antibodies for RORγ (B2D), Gr1 (RB6-8C5) and IL-4 (11B11) were purchased from eBioscience. FITC Annexin V Apoptosis Detection Kit I (2293683) was purchased from BD Phamingen. Anti-RORγt, anti-T-bet (MBL), anti-Thymidylate Synthase, anti-STAT3, anti-pSTAT3, anti-STAT4 (Cell Signaling), anti-Caspase 1 (Millipore), anti-HA and anti-β-actin (Sigma) antibodies for western blotting were used according to the manufacturers’ instructions. Secondary antibodies were from Santa Cruz Biotechnology, Inc.

### CD4^+^ T cell preparation and differentiation *in vitro*

Naive CD4^+^ T cells (CD62L^+^CD44^lo^) were prepared by fluorescence-activated cell sorting from spleens and lymph nodes of C57BL/6 mice. The sorted cells were primed for 96 hrs with anti-CD3 (1 μg/ml; 145-2C11; BD Biosciences) and soluble anti-CD28 (2 μg/ml; 37.51; BD Biosciences). Cells stimulated under neutral conditions were defined as T_H_0 cells. Cells were stimulated to differentiate into T_H_1 cells by supplementation with IL-12 plus anti-IL-4 or into T_H_2 cells by supplementation with IL-4 and anti-IFN-γ. For T_H_17 cell differentiation, cells were stimulated with transforming growth factor-β1 (5 ng/ml), IL-6 (20 ng/ml) and IL-23 (10 ng/ml; all from R&D Systems) and into Treg cells by supplementation with transforming growth factor-β1 (15 ng/ml).

### Preparation of bone marrow derived macrophages

Bone marrow (BM) cells were isolated from tibias and femurs of C57BL/6 mice and the cells were cultured in complete DMEM supplemented with GM-CSF (10 ng/ml). On day 6 or 7, bone marrow derived macrophages (BMDMs) were harvested and then seeded in fresh complete DMEM medium at a density of 2 × 10^6^ cells/ml for experiments.

### Intracellular staining and flow cytometry

For T cells, cells were stimulated with PMA and ionomycin for 5 hrs in the presence of brefeldin A prior to intracellular staining. Cells were fixed with IC Fixation Buffer (BD Bioscience), incubated with permeabilization buffer, and stained with antibodies. For macrophages, bone marrow derived macrophages were activated with LPS (200 ng/ml) plus IFN-gamma (10 ng/ml) overnight and brefeldin A was added to the culture for 5 hrs prior to intracellular staining. Flow cytometry was performed on a FACS Calibur (BD Biosciences).

### Transfection and luciferase reporter assay

For each transfection, 2.0 μg of plasmid was mixed with 100 μl of DMEM (without serum and antibiotics) and 4.0 μl of Lipofectamine^TM^ 2000 reagent. The mixture was incubated at room temperature for 20 minutes and added to 12-well plates containing cells and complete medium. The cells were incubated for 30 hrs and harvested using reporter lysis buffer (Promega) for determination of luciferase activity. Cells were co-transfected with a β-galactosidase reporter plasmid to normalize experiments for transfection efficiency.

### Chromatin immunoprecipitation (ChIP) assay

The ChIP procedure was performed using an assay kit following the manufacturer's instruction (EMD Millipore). Briefly, T_H_17 and T_H_1 cells were cross-linked by 1% formaldehyde for 10 min at 37^°^C. Nuclei were prepared and subjected to sonication to obtain DNA fragments. Chromatin fractions were precleared with protein A-agarose beads followed by immunoprecipitation overnight at 4^°^C with 3 μg of anti-STAT3 (Santa Cruz), anti-ROR gamma(t) (eBioscience), anti-STAT4 (Santa Cruz), or control antibody. Cross-linking was reversed at 65^°^C for 4 hrs, followed by proteinase K digestion. DNA was purified and subjected to qPCR. The input DNA was diluted 200 times prior to PCR amplification. The input and immunoprecipitated DNA were amplified by qPCR using primers encompassing the STAT3 or STAT4 binding sites of the mouse RORγt or T-bet promoter regions.

### Thymidylate synthase SiRNA

Thymidylate Synthase Accell SMART pool small interfering RNA (siRNA) was transfected into cells according to manufacturer's (Dharmacon, Thermo Scientific) protocol. Briefly, Thymidylate Synthase Accell SMART pool siRNA was introduced to murine T cells in the Accell delivery medium. Delivery efficiency and siRNA specificity were tested by using Accell green (FITC) nontargeting control siRNA, GAPDH-specific siRNA, and an Accell nontargeting control siRNA (Dharmacon).

### Cytokine ELISA

Supernatants from cell cultures were collected after activation under various conditions and secreted cytokines in the supernatants were measured by ELISA kits with purified coating and biotinylated detection antibodies: anti-IL-17, anti-IFN-γ (R & D systems), anti-IL-9, anti-IL-22, anti-IL-1β (e Bioscience) and anti-IL-4 (BD Bioscience).

### RNA isolation and quantitative real-time RT-PCR (qPCR)

Total RNA was extracted using an RNeasy plus kit (QIAGEN, Valencia, CA) and cDNA was generated with an oligo (dT) primer and the Superscript II system (Invitrogen, USA) followed by analysis using iCycler PCR with SYBR Green PCR master Mix (Applied Biosystems). Results were normalized based on the expression of ubiquitin. The sequences of primers are shown in Supplementary Table 1.

### T cell-transfer colitis

T cell transfer colitis was performed as previously described [48, 49]. Briefly, purified CD4^+^CD45RB^hi^ T cells from WT mice were injected intraperitoneally into *Rag1*^−/−^ recipients (5 × 10^5^ cells per mouse in 200 μl sterile PBS per injection). Mice were weighed every week throughout the course of experiments. The degree of inflammation in the epithelium, submucosa and submuscularis propria was scored separately as described previously [48].

### Statistical analysis

The results are shown as means ± SD and statistical analysis was performed using Student's *t*-Test. Where more than two groups were compared, one way- ANOVA with Bonferroni's correction were performed. *p* < 0.05 were considered statistically significant.

## SUPPLEMENTARY MATERIAL TABLE AND FIGURES



## References

[1] Korn T, Bettelli E, Oukka M, Kuchroo VK (2009). IL-17 and Th17 Cells. Annu Rev Immunol.

[2] Weaver CT, Harrington LE, Mangan PR, Gavrieli M, Murphy KM (2006). Th17: an effector CD4 T cell lineage with regulatory T cell ties. Immunity.

[3] Mangan PR, Harrington LE, O'Quinn DB, Helms WS, Bullard DC, Elson CO, Hatton RD, Wahl SM, Schoeb TR, Weaver CT (2006). Transforming growth factor-beta induces development of the T(H)17 lineage. Nature.

[4] Bettelli E, Carrier Y, Gao W, Korn T, Strom TB, Oukka M, Weiner HL, Kuchroo VK (2006). Reciprocal developmental pathways for the generation of pathogenic effector TH17 and regulatory T cells. Nature.

[5] O'Garra A, Stockinger B, Veldhoen M (2008). Differentiation of human T(H)-17 cells does require TGF-beta!. Nat Immunol.

[6] Bettelli E, Oukka M, Kuchroo VK (2007). T(H)-17 cells in the circle of immunity and autoimmunity. Nat Immunol.

[7] Korn T, Bettelli E, Gao W, Awasthi A, Jager A, Strom TB, Oukka M, Kuchroo VK (2007). IL-21 initiates an alternative pathway to induce proinflammatory T(H)17 cells. Nature.

[8] Nurieva R, Yang XO, Martinez G, Zhang Y, Panopoulos AD, Ma L, Schluns K, Tian Q, Watowich SS, Jetten AM, Dong C (2007). Essential autocrine regulation by IL-21 in the generation of inflammatory T cells. Nature.

[9] Yang L, Anderson DE, Baecher-Allan C, Hastings WD, Bettelli E, Oukka M, Kuchroo VK, Hafler DA (2008). IL-21 and TGF-beta are required for differentiation of human T(H)17 cells. Nature.

[10] Dong C (2008). TH17 cells in development: an updated view of their molecular identity and genetic programming. Nat Rev Immunol.

[11] Bettelli E, Korn T, Kuchroo VK (2007). Th17: the third member of the effector T cell trilogy. Curr Opin Immunol.

[12] Zheng Y, Danilenko DM, Valdez P, Kasman I, Eastham-Anderson J, Wu J, Ouyang W (2007). Interleukin-22, a T(H)17 cytokine, mediates IL-23-induced dermal inflammation and acanthosis. Nature.

[13] Liang SC, Tan XY, Luxenberg DP, Karim R, Dunussi-Joannopoulos K, Collins M, Fouser LA (2006). Interleukin (IL)-22 and IL-17 are coexpressed by Th17 cells and cooperatively enhance expression of antimicrobial peptides. J Exp Med.

[14] Ivanov II, McKenzie BS, Zhou L, Tadokoro CE, Lepelley A, Lafaille JJ, Cua DJ, Littman DR (2006). The orphan nuclear receptor RORgammat directs the differentiation program of proinflammatory IL-17+ T helper cells. Cell.

[15] Gu Y, Yang J, Ouyang X, Liu W, Li H, Bromberg J, Chen SH, Mayer L, Unkeless JC, Xiong H (2008). Interleukin 10 suppresses Th17 cytokines secreted by macrophages and T cells. Eur J Immunol.

[16] Brustle A, Heink S, Huber M, Rosenplanter C, Stadelmann C, Yu P, Arpaia E, Mak TW, Kamradt T, Lohoff M (2007). The development of inflammatory T(H)-17 cells requires interferon-regulatory factor 4. Nat Immunol.

[17] Byersdorfer CA, Tkachev V, Opipari AW, Goodell S, Swanson J, Sandquist S, Glick GD, Ferrara JL (2013). Effector T cells require fatty acid metabolism during murine graft-*versus*-host disease. Blood.

[18] Dang EV, Barbi J, Yang HY, Jinasena D, Yu H, Zheng Y, Bordman Z, Fu J, Kim Y, Yen HR, Luo W, Zeller K, Shimoda L, Topalian SL, Semenza GL, Dang CV (2011). Control of T(H)17/T(reg) balance by hypoxia-inducible factor 1. Cell.

[19] Kleinewietfeld M, Manzel A, Titze J, Kvakan H, Yosef N, Linker RA, Muller DN, Hafler DA (2013). Sodium chloride drives autoimmune disease by the induction of pathogenic TH17 cells. Nature.

[20] Sundrud MS, Koralov SB, Feuerer M, Calado DP, Kozhaya AE, Rhule-Smith A, Lefebvre RE, Unutmaz D, Mazitschek R, Waldner H, Whitman M, Keller T, Rao A (2009). Halofuginone inhibits TH17 cell differentiation by activating the amino acid starvation response. Science.

[21] Yen D, Cheung J, Scheerens H, Poulet F, McClanahan T, McKenzie B, Kleinschek MA, Owyang A, Mattson J, Blumenschein W, Murphy E, Sathe M, Cua DJ, Kastelein RA, Rennick D (2006). IL-23 is essential for T cell-mediated colitis and promotes inflammation *via* IL-17 and IL-6. The Journal of clinical investigation.

[22] Leppkes M, Becker C, Ivanov II, Hirth S, Wirtz S, Neufert C, Pouly S, Murphy AJ, Valenzuela DM, Yancopoulos GD, Becher B, Littman DR, Neurath MF (2009). RORgamma-expressing Th17 cells induce murine chronic intestinal inflammation *via* redundant effects of IL-17A and IL-17F. Gastroenterology.

[23] Fuss IJ, Becker C, Yang Z, Groden C, Hornung RL, Heller F, Neurath MF, Strober W, Mannon PJ (2006). Both IL-12p70 and IL-23 are synthesized during active Crohn's disease and are down-regulated by treatment with anti-IL-12 p40 monoclonal antibody. Inflammatory bowel diseases.

[24] O'Connor W, Kamanaka M, Booth CJ, Town T, Nakae S, Iwakura Y, Kolls JK, Flavell RA (2009). A protective function for interleukin 17A in T cell-mediated intestinal inflammation. Nat Immunol.

[25] Fitzpatrick LR (2013). Inhibition of IL-17 as a pharmacological approach for IBD. International reviews of immunology.

[26] Heidelberger C, Chaudhuri NK, Danneberg P, Mooren D, Griesbach L, Duschinsky R, Schnitzer RJ, Pleven E, Scheiner J (1957). Fluorinated pyrimidines, a new class of tumour-inhibitory compounds. Nature.

[27] Longley DB, Harkin DP, Johnston PG (2003). 5-fluorouracil: mechanisms of action and clinical strategies. Nature reviews Cancer.

[28] Wilke CM, Bishop K, Fox D, Zou W (2011). Deciphering the role of Th17 cells in human disease. Trends Immunol.

[29] Fonville NC, Vaksman Z, DeNapoli J, Hastings PJ, Rosenberg SM (2011). Pathways of resistance to thymineless death in Escherichia coli and the function of UvrD. Genetics.

[30] Houghton JA, Harwood FG, Tillman DM (1997). Thymineless death in colon carcinoma cells is mediated *via* fas signaling. Proceedings of the National Academy of Sciences of the United States of America.

[31] Nakaya M, Xiao Y, Zhou X, Chang JH, Chang M, Cheng X, Blonska M, Lin X, Sun SC (2014). Inflammatory T cell responses rely on amino acid transporter ASCT2 facilitation of glutamine uptake and mTORC1 kinase activation. Immunity.

[32] Souglakos J, Androulakis N, Syrigos K, Polyzos A, Ziras N, Athanasiadis A, Kakolyris S, Tsousis S, Kouroussis C, Vamvakas L, Kalykaki A, Samonis G, Mavroudis D, Georgoulias V (2006). FOLFOXIRI (folinic acid, 5-fluorouracil, oxaliplatin and irinotecan) *vs* FOLFIRI (folinic acid, 5-fluorouracil and irinotecan) as first-line treatment in metastatic colorectal cancer (MCC): a multicentre randomised phase III trial from the Hellenic Oncology Research Group (HORG). British journal of cancer.

[33] Falcone A, Ricci S, Brunetti I, Pfanner E, Allegrini G, Barbara C, Crino L, Benedetti G, Evangelista W, Fanchini L, Cortesi E, Picone V, Vitello S, Chiara S, Granetto C, Porcile G (2007). Phase III trial of infusional fluorouracil, leucovorin, oxaliplatin, and irinotecan (FOLFOXIRI) compared with infusional fluorouracil, leucovorin, and irinotecan (FOLFIRI) as first-line treatment for metastatic colorectal cancer: the Gruppo Oncologico Nord Ovest. Journal of clinical oncology.

[34] Neoptolemos JP, Moore MJ, Cox TF, Valle JW, Palmer DH, McDonald AC, Carter R, Tebbutt NC, Dervenis C, Smith D, Glimelius B, Charnley RM, Lacaine F, Scarfe AG, Middleton MR, Anthoney A (2012). Effect of adjuvant chemotherapy with fluorouracil plus folinic acid or gemcitabine *vs* observation on survival in patients with resected periampullary adenocarcinoma: the ESPAC-3 periampullary cancer randomized trial. Jama.

[35] Cooper HS, Murthy S, Kido K, Yoshitake H, Flanigan A (2000). Dysplasia and cancer in the dextran sulfate sodium mouse colitis model. Relevance to colitis-associated neoplasia in the human: a study of histopathology, B-catenin and p53 expression and the role of inflammation. Carcinogenesis.

[36] Cohen EE, Karrison TG, Kocherginsky M, Mueller J, Egan R, Huang CH, Brockstein BE, Agulnik MB, Mittal BB, Yunus F, Samant S, Raez LE, Mehra R, Kumar P, Ondrey F, Marchand P (2014). Phase III randomized trial of induction chemotherapy in patients with N2 or N3 locally advanced head and neck cancer. Journal of clinical oncology.

[37] Chang CH, Curtis JD, Maggi LB, Faubert B, Villarino AV, O'sullivan D, Huang SC, van der Windt GJ, Blagih J, Qiu J, Weber JD, Pearce EJ, Jones RG, Pearce EL (2013). Posttranscriptional control of T cell effector function by aerobic glycolysis. Cell.

[38] Jacobs SR, Herman CE, Maciver NJ, Wofford JA, Wieman HL, Hammen JJ, Rathmell JC (2008). Glucose uptake is limiting in T cell activation and requires CD28-mediated Akt-dependent and independent pathways. Journal of immunology.

[39] Tannahill GM, Curtis AM, Adamik J, Palsson-McDermott EM, McGettrick AF, Goel G, Frezza C, Bernard NJ, Kelly B, Foley NH, Zheng L, Gardet A, Tong Z, Jany SS, Corr SC, Haneklaus M (2013). Succinate is an inflammatory signal that induces IL-1beta through HIF-1alpha. Nature.

[40] Yan Z, Garg SK, Banerjee R (2010). Regulatory T cells interfere with glutathione metabolism in dendritic cells and T cells. The Journal of biological chemistry.

[41] Ghoshal K, Jacob ST (1994). Specific inhibition of pre-ribosomal RNA processing in extracts from the lymphosarcoma cells treated with 5-fluorouracil. Cancer research.

[42] Houghton JA, Tillman DM, Harwood FG (1995). Ratio of 2′-deoxyadenosine-5′-triphosphate/thymidine-5′-triphosphate influences the commitment of human colon carcinoma cells to thymineless death. Clinical cancer research.

[43] Santi DV, Hardy LW (1987). Catalytic mechanism and inhibition of tRNA (uracil-5-)methyltransferase: evidence for covalent catalysis. Biochemistry.

[44] Ghiringhelli F, Apetoh L, Tesniere A, Aymeric L, Ma Y, Ortiz C, Vermaelen K, Panaretakis T, Mignot G, Ullrich E, Perfettini JL, Schlemmer F, Tasdemir E, Uhl M, Genin P, Civas A (2009). Activation of the NLRP3 inflammasome in dendritic cells induces IL-1beta-dependent adaptive immunity against tumors. Nature medicine.

[45] Vincent J, Mignot G, Chalmin F, Ladoire S, Bruchard M, Chevriaux A, Martin F, Apetoh L, Rebe C, Ghiringhelli F (2010). 5-Fluorouracil selectively kills tumor-associated myeloid-derived suppressor cells resulting in enhanced T cell-dependent antitumor immunity. Cancer research.

[46] Kanterman J, Sade-Feldman M, Biton M, Ish-Shalom E, Lasry A, Goldshtein A, Hubert A, Baniyash M (2014). Adverse immunoregulatory effects of 5FU and CPT11 chemotherapy on myeloid-derived suppressor cells and colorectal cancer outcomes. Cancer research.

[47] Bruchard M, Mignot G, Derangere V, Chalmin F, Chevriaux A, Vegran F, Boireau W, Simon B, Ryffel B, Connat JL, Kanellopoulos J, Martin F, Rebe C, Apetoh L, Ghiringhelli F (2013). Chemotherapy-triggered cathepsin B release in myeloid-derived suppressor cells activates the Nlrp3 inflammasome and promotes tumor growth. Nature medicine.

[48] Totsuka T, Kanai T, Nemoto Y, Makita S, Okamoto R, Tsuchiya K, Watanabe M (2007). IL-7 Is essential for the development and the persistence of chronic colitis. Journal of immunology.

[49] Powrie F, Leach MW, Mauze S, Caddle LB, Coffman RL (1993). Phenotypically distinct subsets of CD4+ T cells induce or protect from chronic intestinal inflammation in C. B-17 scid mice. International immunology.

[50] Pearson JS, Giogha C, Ong SY, Kennedy CL, Kelly M, Robinson KS, Lung TW, Mansell A, Riedmaier P, Oates CV, Zaid A, Muhlen S, Crepin VF, Marches O, Ang CS, Williamson NA (2013). A type III effector antagonizes death receptor signalling during bacterial gut infection. Nature.

