# Supplementary material for: 5-Fluorouracil targets thymidylate synthase in the selective suppression of TH17 cell differentiation
Source: Oncotarget. 2016 Mar 24;7(15):19312–26. doi: 10.18632/oncotarget.8344 (PMC4991385; doi:10.18632/oncotarget.8344)
Supplement: Supplementary file 1 [file oncotarget-07-19312-s001.pdf]

5-Fluorouracil targets thymidylate synthase in the selective suppression of T_H17 cell differentiation

Supplementary Material

Supplementary Table 1: Oligonucleotide sequences used in quantitative RT-PCR analysis.

Gene	Forward(5'→ 3')	Reverse(5'→ 3')
IL-17A	CTCCAGAAGGCCCTCAGACTAC	AGCTTTCCCTCCGCATTGACACAG
RORγt	CCGCTGAGAGGGCTTCAC	TGCAGGAGTAGGCCACATTACA
IFN-γ	ACTGGCAAAAGGATGGTG	GTTGCTGATGGCCTGATT
T-bet	CCTGGACCCAACTGTCAACT	AACTGTGTTCCCGAGGTGTG
IL-4	GGCATTTTGAACGAGGTCACA	CTCACCGAGCTCTGTTGACAAG
Foxp3	CCCAGGAAAGACAGCAACCTT	TTCTCACAACCAGGCCACTTG
TS	CAATGGATCCCGAGATTTTC	GTCATCAGGGTTGGTTTTGA
GATA-3	GAAGGCATCCAGACCCGAAAC	ACCCATGGCGGTGACCATGC
IL-10	TTTGAATTCCCTGGGTGAGAA	GCTCCACTGCCTTGCTC
TGF-β	GCC-ACCACGCTCTTCTGTCT	GGTCTGGGCCATAGAACTGATG
IL-9	CATCAGTGTCTCTCCGTCCCAACTGATG	GATTTCTGTGTGG CATTGGTCAG
IL-22	CATGCAGGAGGTGGTACCTT	CAGACGCAAGCATTTCTCAG
TNF-α	GCCACCACGCTCTTCTGTCT	GGTCTGGGCCATAGAACTGATG
IL-1β	GGAGAACCAAGCAACGACAAAATA	TGGGGAACTCTGCAGACTCAAAC
NLRP3	AGAGCCTACAGTTGGGTGAAATG	CCACGCCTACCAGGAAATCTC
Ubiquitin	TGGCTATTAATTATTCGGTCTGCA	GCAAGTGGCTAGAGTGCAGAGTAA

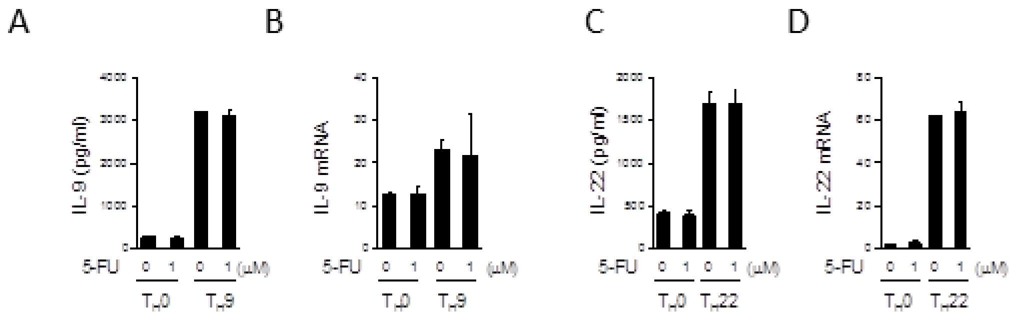

Supplementary Figure 1. Low dose 5-FU has no significant effect in suppressing T_H9 and T_H22 cell differentiation. (A) Naïve $CD4^+$ T cells from C57BL/6 mice were differentiated under T_H9 polarizing conditions in the presence of 5-FU (1.0 μM) for 3 days and the supernatants were analyzed for IL-9 by ELISA. Each bar represents mean ± SD from three independent experiments. (B) The cells prepared in (A) were cultured for 48 hours and IL-9 mRNA expression was determined by qPCR. (C) Naïve $CD4^+$ T cells from C57BL/6 mice were differentiated under T_H22 polarizing conditions in the presence of 5-FU (1.0 μM) for 3 days, and the supernatants were analyzed for IL-22 by ELISA.(D) The cells prepared in (C) were cultured for 48 hours and IL-22 mRNA expression was determined by qPCR. Each bar represents mean ± SD from three independent experiments.

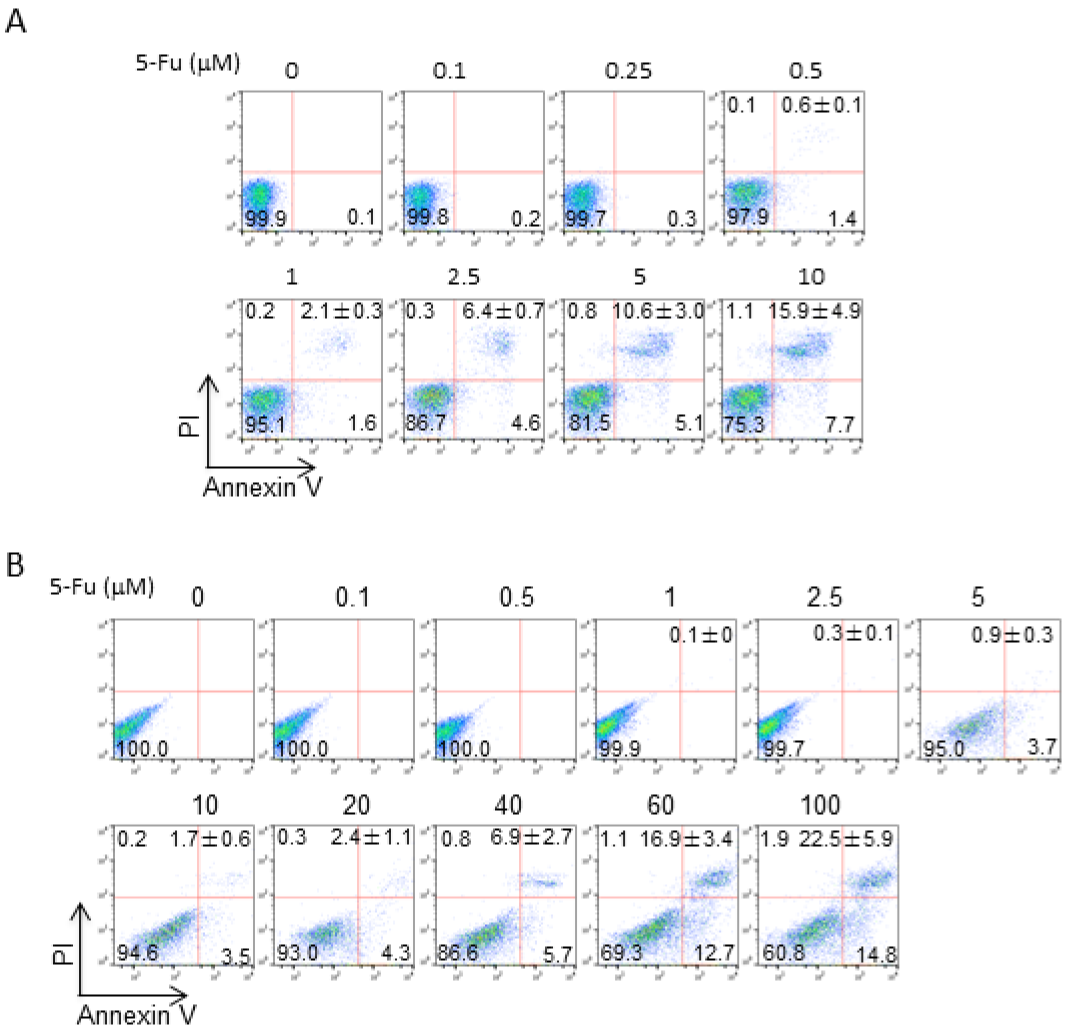

Supplementary Figure 2. Low dose 5-FU does not induce T_H17 or SW620 cell apoptosis. (A) Apoptosis of T_H17 cells treated with different concentrations of 5-FU was assessed using AnnexinV/PI staining and flow cytometry. (B) Apoptosis of SW620 cells treated with different concentrations of 5-FU was assessed using Annexin V/PI staining and flow cytometry.

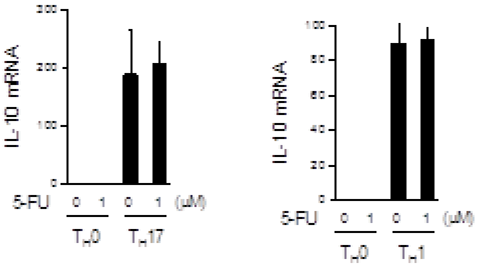

Supplementary Figure 3. Low dose 5-FU does not suppress IL-10 expression of T_H17 or T_H1 cell. Naïve $CD4^+$ T cells from C57BL/6 mice were differentiated under T_H17 and T_H1 polarizing conditions respectively in the presence of 5-FU (1.0 μM) for 48 hours; IL-10 mRNA expression was determined by qPCR. Each bar represents mean ± SD from three independent experiments.

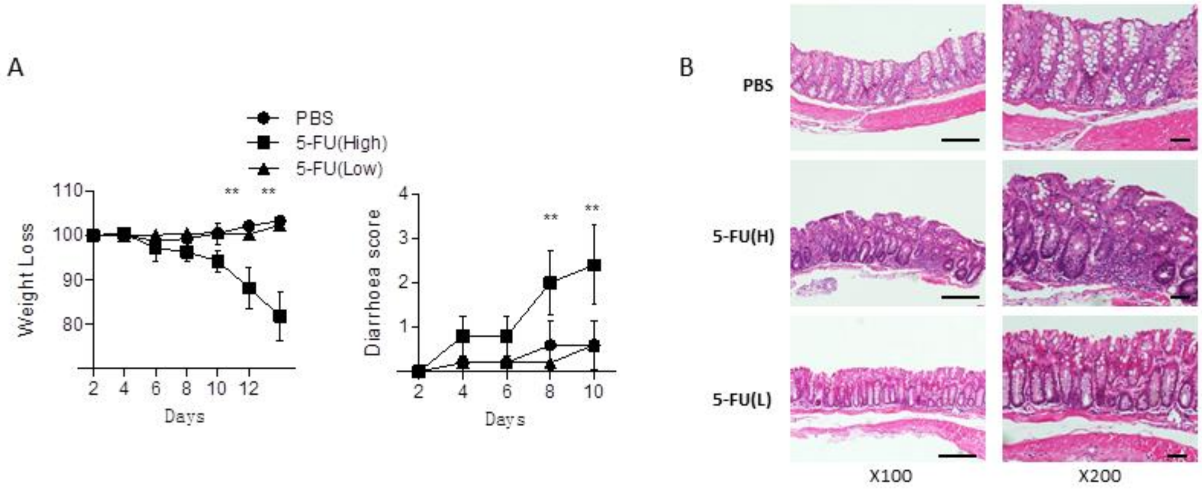

Supplementary Figure 4. Low dose 5-FU has no significant toxicity on the intestine mucosa in mice. C57BL/6 mice were injected (i.p.) with 5-FU (50 mg/kg, 10 mg/kg) or PBS every three days for 2 weeks. Body weight and diarrhea scores[50] were recorded. (A) Weight changes and diarrheae scores during treatment were shown from day 0. **p< 0.01 versus recipients of PBS treated group. (B) Sections of colons with colitis were shown. Scale bar, 100 μm.

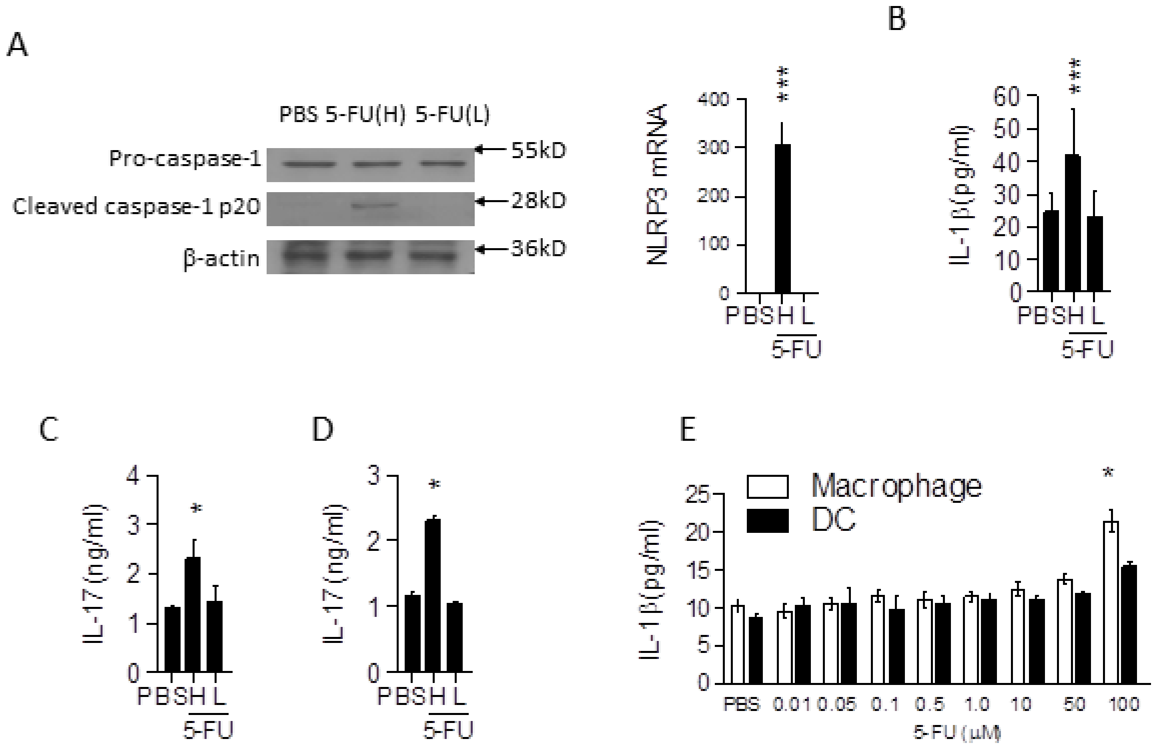

Supplementary Figure 5. Low dose 5-FU does not promote the secretion of bioactive IL-1β in myeloid cells . (A) LLC tumor-bearing mice were treated (i.p.) with 5-FU (50mg/kg, 10mg/kg) or PBS for 48 hours and splenic MDSCs were isolated. Then caspase-1 protein expression were analyzed by western blotting, and NLRP3 mRNA expression were analyzed by qPCR, each bar represents mean ± SD of measurements made from five mice, ***p< 0.001 versus recipients of PBS treated group. (B) the splentic MDSCs prepared in (A) were cultured for 3 days and the supernatants were analyzed by ELISA for IL-1β seretion, each bar represents mean ± SD of measurements made from five mice, *p< 0.05 versus recipients of PBS treated group. (C) The cells prepared in (A) cultured with naïve $CD4^+CD62L^+$ T cells and activated in the presence of antibodies to CD3 (5μg ml^{-1}) and CD28 (5μg ml^{-1}) for 3 days and the supernatants were analyzed by ELISA for IL-17 seretion, each bar represents mean ± SD of measurements made from five

mice, *p< 0.05 versus recipients of PBS treated group. (D) LLC tumor-bearing mice were treated (i.p.) with 5-FU (50mg/kg,10mg/kg) or PBS for 5 days and draining lymph node cells were harvested , then the cells were cultured in the presence of antibodies to CD3(5μg ml^{-1}) and CD28(5μg ml^{-1}) for 3 days. The supertants of the cells were analyzed by ELISA for IL-17 seretion, each bar represents mean ± SD of measurements made from five mice, *p< 0.05 versus recipients of PBS treated group. (E) BMDMs from C57BL/6 mice were stimulated with IFN-gamma (10 ng/ml) plus LPS (200 ng/ml) or GM-CSF(20 ng/ml) plus IL-4(10 ng/ml) in the presence of 5-FU at different concentrations for 24 hours, the supernatants of the cells were analyzed for IL-1β by ELISA, Each bar represents mean ± SD from three independent experiments. *p< 0.05 versus cells treated with PBS.
